# Simulation Evaluation for Methods Used to Determine Muscular Internal Force Based on Joint Stiffness Using Muscular Internal Force Feedforward Controller for Musculoskeletal System

**DOI:** 10.3389/frobt.2021.699792

**Published:** 2021-09-27

**Authors:** Yuki Matsutani, Kenji Tahara, Hitoshi Kino

**Affiliations:** ^1^ Department of Robotics, Faculty of Engineering, Kindai University, Higashi-Hiroshima, Japan; ^2^ Department of Mechanical Engineering, Faculty of Engineering, Kyushu University, Fukuoka, Japan; ^3^ School of Engineering, Chukyo University, Nagoya, Japan

**Keywords:** musculoskeletal system, feedforward controller, joint stiffness, redundancy, internal force

## Abstract

This study proposes two novel methods for determining the muscular internal force (MIF) based on joint stiffness, using an MIF feedforward controller for the musculoskeletal system. The controller was developed in a previous study, where we found that it could be applied to achieve any desired end-point position without the use of sensors, by providing the MIF as a feedforward input to individual muscles. However, achieving motion with good response and low stiffness using the system, posed a challenge. Furthermore, the controller was subject to an ill-posed problem, where the input could not be uniquely determined. We propose two methods to improve the control performance of this controller. The first method involves determining a MIF that can independently control the response and stiffness at a desired position, and the second method involves the definition of an arbitrary vector that describes the stiffnesses at the initial and desired positions to uniquely determine the MIF balance at each position. The numerical simulation results reported in this study demonstrate the effectiveness of both proposed methods.

## 1 Introduction

Humans are able to easily achieve specific complex movements by manipulating the structure of the human body. The human body structure can be described in terms of a musculoskeletal structure in which muscles, corresponding to actuators, cover bones and joints. Joint angle and stiffness can be adjusted by contracting the muscles, thus allowing the individual to perform complex motions.

A number of robots that can imitate this musculoskeletal structure have recently been investigated. Marques et al. (Marques et al., 2010) developed a human coexistence robot that imitates the musculoskeletal structure, while a research group including Inaba (Mizuuchi et al., 2006; Asano et al., 2016) developed another musculoskeletal structure-imitating humanoid robot. Verrelst et al. (Verrelst et al., 2005) developed a biped robot actuated with antagonistic pneumatic artificial muscles. This robot can control joint stiffness and achieve stable walking. Niiyama et al. (Niiyama et al., 2012) developed a double legged robot with a musculoskeletal structure that emulates an athlete’s physique and style of running. Their robot had the capability of running stably for 4 m. Niiyama et al. (Niiyama et al., 2007) developed a jumping robot with a musculoskeletal structure that could perform big leaps. The results of these studies indicate that the musculoskeletal structure functions as a feedback system when undergoing instantaneous movements; thus, an understanding of the musculoskeletal structure can be used to develop robots that can perform previously difficult-to-achieve motions.

In a previous study, we proposed a muscular internal force (MIF) feedforward controller for a musculoskeletal system (Kino et al., 2009, 2013). This controller could be used to attain any desired position without the use of sensors, by providing a MIF as a feedforward input to the associated muscle. The controller input would create a potential field in the musculoskeletal system; allowing the system to converge at the desired position when this potential field achieved a stable equilibrium point at the desired position. This end-point convergence is influenced by muscle arrangement (Kino et al., 2017). The MIF must be increased to obtain a desirable control response, through increase in system stiffness. Subsequently, reducing the MIF to lower the stiffness causes the responsiveness to deteriorate. As a result of the correlation between response and stiffness, it is challenging to simultaneously achieve high-response motion and low system stiffness. Furthermore, the controller is affected by an ill-posed problem where, its input cannot be uniquely determined due to the muscle redundancy of the musculoskeletal system.

To tackle this muscle redundancy, Buchler et al. (Buchler et al., 2018) proposed a variance-regularized control that can achieve high accuracy trajectory tracking in the musculoskeletal system. In addition, Jantsch et al. (Jantsch et al., 2011) proposed a new controller based on computed torque control and a method for obtaining the muscle Jacobian by using machine-learning techniques for the musculoskeletal system. Their method indicates that trajectory control and force control can be achieved. Katayama and Kawato (Katayama and Kawato, 1990) proposed a parallel-hierarchical neural network model based on a feedback-error-learning scheme, in the field of exercise physiology. This model requires repeated trials; however, it is capable of achieving rapid movements that have otherwise been difficult to achieve using a feedback controller. A tendon driven robot equivalent to the musculoskeletal system has been explored (Viau et al., 2015). However, position control methods, like the feedforward controller method from our previous study (Kino et al., 2009, 2013), have not been fully explored.

Further, various studies have been conducted on methods for contributive employment of redundancies (Klein and Huang, 1983). For instance, it has been observed that redundancy can be used to avoid specific robot attitudes (Mayorga and Wong, 1988) and obstacles (Baillieul, 1986; Chirikjian and Burdick, 1990) during motion. In addition, redundancies can be used to optimize torques in the robotic structure (Suh and Hollerbach, 1987), avoid restrictive joint movement (Shimizu et al., 2008), and apply stiffness (Svinin et al., 2002) and impedance control (Albu-Schaffer et al., 2003). Yoshikawa (Yoshikawa, 1985) suggested that the operability of a manipulator can be improved using redundancy; while, Hanafusa et al. (Hanafusa et al., 1981) and Nakamura et al. (Nakamura and Hanafusa, 1987) proposed control methods for achieving sub-tasks, based on the prioritization of robot actions. In parallel wire-driven systems (Sui and Zhao, 2004; Behzadipour and Khajepour, 2006), system actuation redundancy can be used to generate internal wire forces. The stiffness of the system can be changed by controlling these internal forces, thus indicating that robot performance and versatility can be improved by manipulating the robot’s redundancy. Hence, redundancy has several potential advantages. Meanwhile, sensors are mostly utilized in methods that employ redundancy. The musculoskeletal system continues to encounter muscle redundancy. The musculoskeletal system can achieve various kinds of motion by utilizing muscle redundancy without the addition of a new actuator.

The primary objective of this study is, to resolve the issues in the MIF feedforward controller proposed in previous studies. We began by eliminating the foremost issue of correlation between the response and stiffness. Further, the MIF that separately sets the response and stiffness was determined, and the ill-posed problem was solved. In this study, a new generic method for determining MIF using joint stiffness was developed, as an approach to improving the control performance of our previously developed MIF feedforward controller. The proposed method involves the application of an approach derived from Nakamura (Nakamura and Hanafusa, 1987), which includes prioritized application of sub-tasks to increase the stiffness of joints at desired positions by way of redundancy. This approach was used to develop two specific MIF determination methods: one involving the independent setting of response and stiffness, and the other involving the unique determination of MIF. The first method was used to identify the MIF that can independently determine the response and stiffness at a desired position, while the second was used to determine an arbitrary vector that reflects the stiffness at the initial and desired positions, and uniquely defines the balance of MIFs at each position.

The remainder of this paper is organized as follows. Section 2 describes the kinematics of the musculoskeletal system. Section 3 describes the MIF feedforward system used to control the musculoskeletal system. In Section 4, describes the development of a joint stiffness matrix of the musculoskeletal system. The two methods and their simulation results are presented in Sections 5 and Section 6, respectively.

## 2 Musculoskeletal System

**FIGURE 1:**
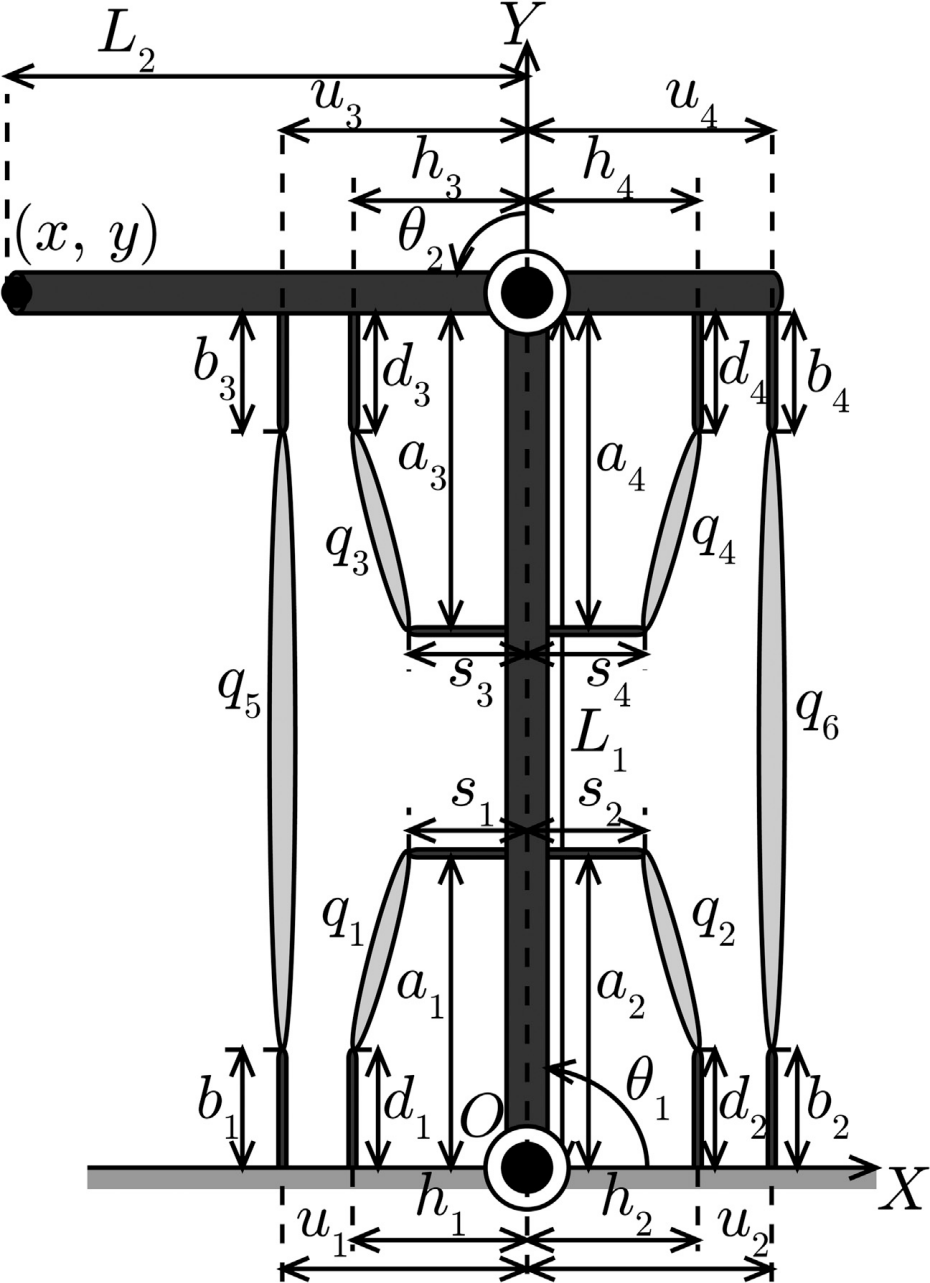
Musculoskeletal system.

The musculoskeletal system considered in this study is shown in Figure 1. The system comprised of two links and six muscles. The joint had one degree of freedom and could rotate when muscle tension was applied through the link. A joint with one degree of freedom requires at least two muscles to drive its link in the clockwise and counter-clockwise directions because muscles can only produce shrinkage forces; as a result of which, this system has an actuation redundancy. Additionally, we assumed that the movement of the system was unaffected by the force of gravity because the system was set in the *x*-*y* plane.

The muscle consisted of a combination of wire and motor instead of a pneumatic actuator (Kino et al., 2017). The muscles were connected to an anchored base, and to each link. The anchoring was set at an explicit offset that influenced the convergence of the MIF feedforward controller (Kino et al., 2009, 2013). In our analysis of the muscular arrangement, we employed a set of parameters that converged at a desired position. Although, as mentioned above, the influence of gravity is also not considered; however, the muscular internal force feedforward controller is able to achieve system movement under the influence of gravity from a result of an other study (Matsutani et al., 2019b).

### 2.1 Relation Between Muscle and Joint Spaces

The relation between the muscle length vector $q\left(\theta \right)={\left[q_{1},\ldots ,q_{6}\right]}^{T}$ and the joint angle vector $\theta ={\left[\theta_{1},\theta_{2}\right]}^{T}$ within the musculoskeletal system is given by

$$q\left(\theta \right)=\left[\begin{array}{c} \sqrt{{\left(h_{1}+a_{1}C_{1}-s_{1}S_{1}\right)}^{2}+{\left(d_{1}-a_{1}S_{1}-s_{1}C_{1}\right)}^{2}}\\ \sqrt{{\left(h_{2}-a_{2}C_{1}-s_{2}S_{1}\right)}^{2}+{\left(d_{2}-a_{2}S_{1}+s_{2}C_{1}\right)}^{2}}\\ \sqrt{{\left(h_{3}+a_{3}C_{2}-s_{3}S_{2}\right)}^{2}+{\left(d_{3}-a_{3}S_{2}-s_{3}C_{2}\right)}^{2}}\\ \sqrt{{\left(h_{4}-a_{4}C_{2}-s_{4}S_{2}\right)}^{2}+{\left(d_{4}-a_{4}S_{2}+s_{4}C_{2}\right)}^{2}}\\ \sqrt{{\left(u_{1}+L_{1}C_{1}+u_{3}C_{12}-b_{3}S_{12}\right)}^{2}+{\left(b_{1}-L_{1}S_{1}-u_{3}S_{12}-b_{3}C_{12}\right)}^{2}}\\ \sqrt{{\left(u_{2}-L_{1}C_{1}+u_{4}C_{12}+b_{4}S_{12}\right)}^{2}+{\left(b_{2}-L_{1}S_{1}+u_{4}S_{12}-b_{4}C_{12}\right)}^{2}} \end{array}\right],$$ (1)

where *L*_*i*_ (*i* = 1, 2) is the link length, *a*_*j*_ (*j* = 1, … , 4), *b*_*j*_, *d*_*j*_, *h*_*j*_, *u*_*j*_, *s*_*j*_ are the muscular arrangement parameters, as shown in Figure 1, *C*_*i*_ and *S*_*i*_ are defined in terms of *i* as *C*_*i*_ = cos *θ*_*i*_ and *S*_*i*_ = sin *θ*_*i*_, respectively, and *C*_12_ and *S*_12_ are defined as *C*_12_ = cos (*θ*_1_ + *θ*_2_) and *S*_12_ = sin (*θ*_1_ + *θ*_2_), respectively. By differentiating Eq. 1 with respect to time, the relation between the muscle contractile vector $\dot{q}\left(\theta \right)\in R^{6}$ and the joint angular velocity vector $\dot{\theta }\in R^{2}$ can be obtained:

$$\dot{q}\left(\theta \right)=-W{\left(\theta \right)}^{T}\dot{\theta },$$ (2)

where $W\left(\theta \right)\in R^{2\times 6}$ is the Jacobi matrix showing the relation between the respective vectors, which is given by

$$W\left(\theta \right)=-\frac{\partial q{\left(\theta \right)}^{T}}{\partial \theta }.$$ (3)

The relation between the joint torque vector $\tau \left(\theta \right)={\left[\tau_{1}\left(\theta \right),\tau_{2}\left(\theta \right)\right]}^{T}$ and the muscle tension vector $\alpha \left(\theta \right)\in R^{6}$ can be obtained based on the principle of virtual work as

τ(θ)=W(θ)α(θ). (4)

The inverse relationship to that given in Eq. 4 is expressed as

$$\begin{array}{c} \alpha \left(\theta \right)=W{\left(\theta \right)}^{+}\tau \left(\theta \right)+\left(I_{6}-W{\left(\theta \right)}^{+}W\left(\theta \right)\right)k_{e}, \end{array}$$ (5)

$$\begin{array}{c} =W{\left(\theta \right)}^{+}\tau \left(\theta \right)+v\left(\theta \right), \end{array}$$ (6)

where $I_{6}\in R^{6\times 6}$ is a unit matrix, $k_{e}\in R^{6}$ is an arbitrary vector, $v\left(\theta \right)\in R^{6}$ is the MIF vector, and $W{\left(\theta \right)}^{+}\in R^{6\times 2}$ is a pseudo inverse matrix of ***W*** (***θ***) defined by

$$W{\left(\theta \right)}^{+}=W{\left(\theta \right)}^{T}{\left(W\left(\theta \right)W{\left(\theta \right)}^{T}\right)}^{-1}.$$ (7)

The MIF vector ***v*** (***θ***) corresponds to the internal force applied within the muscle. This vector belongs to the null space, that is, it is a muscle tension component that cannot generate joint torque and satisfies the following equation

W(θ)v(θ)=0. (8)

As a result, the musculoskeletal system was subjected to an ill-posed problem because the MIF vector corresponding to a given joint torque could not be uniquely determined.

### 2.2 Relation Between Task and Joint Spaces

The relation between the end-point position vector ***x*** (***θ***) = [*x*,*y*]^T^ and the joint angle vector ***θ*** of the musculoskeletal system is given by

$$x\left(\theta \right)=\left[\begin{array}{c} L_{1}C_{1}+L_{2}C_{12}\\ L_{1}S_{1}+L_{2}S_{12} \end{array}\right].$$ (9)

By differentiating Eq. 9 with respect to time, the relation between the end-point velocity vector $\dot{x}\left(\theta \right)\in R^{2}$ and the joint angular velocity vector $\dot{\theta }$ can be obtained as

$$\dot{x}\left(\theta \right)=J\left(\theta \right)\dot{\theta },$$ (10)

Where $J\left(\theta \right)\in R^{2\times 2}$ is the Jacobi matrix showing the relation between the respective vectors.

## 3 Muscular Internal Force Feedforward Controller

The MIF feedforward controller, developed in our previous study (Kino et al., 2009, 2013) was used to positionally control the musculoskeletal system. In this method, the controller inputs a balancing MIF at a desired joint angle of ***θ***_*d*_ into the muscles of the musculoskeletal system. This control input has been expressed using Eqs 5, 6 as

$$\begin{array}{ccc} \alpha \left(\theta \right) & = & v\left(\theta_{d}\right)\\ & = & \left(I_{6}-W{\left(\theta_{d}\right)}^{+}W\left(\theta_{d}\right)\right)k_{e}. \end{array}$$ (11)

Under this method, control was obtained using only kinematics; the input of any physical parameters or sensor information of the musculoskeletal system was not required. The Jacobi matrix ***W*** (***θ***_*d*_) had a constant value at all times, across all joint angular displacements because the matrix was obtained using the desired joint angle ***θ***_*d*_. Additionally, the control input was constant when the arbitrary vector ***k***_***e***_ was constant.

When the shrinkage direction of the muscle was set at a positive, the muscle tension ***α*** (***θ***) also had a positive value because the muscle can produce only tension or shrinkage. Therefore, the elements *v*_*dl*_ (*l* = 1, … , 6) of the MIF $v\left(\theta_{d}\right)={\left[v_{d1},\ldots ,v_{d6}\right]}^{T}$ needed to satisfy the following equation:

$$v_{dl}>0.$$ (12)

Under this control method, the arbitrary vector ***k***_***e***_ was set freely within a range satisfying Eq. 12.

Joint torque cannot be generated at a joint angle of ***θ*** = ***θ***_*d*_ to the muscle, by applying the control input detailed in Eq. 11:

$$W\left(\theta_{d}\right)v\left(\theta_{d}\right)=0.$$ (13)

The control input is a muscle tension component that does not generate joint torque because the MIF vector ***v*** (***θ***_*d*_) belongs to the null space of the Jacobi matrix ***W*** (***θ***_*d*_). By contrast, at joint angles of ***θ*** ≠ ***θ***_*d*_ the control input ***v*** (***θ***_*d*_) cannot be considered to be a vector belonging to the null space of ***W*** (***θ***). It is possible for a control input ***v*** (***θ***_*d*_) to generate a joint torque because ***v*** (***θ***_*d*_) can be decomposed into two components: a muscle tension component that does not generate joint torque and the vector ***v*** (***θ***) that belongs to the null space:

$$W\left(\theta \right)v\left(\theta_{d}\right)=0\;\;\;or\;\;\;W\left(\theta \right)v\left(\theta_{d}\right)\neq 0.$$ (14)

Using the abovementioned characteristics, the MIF controller generated joint movement.

The convergence of the motion end-point was related to the potential *P* generated by the MIF ***v*** (***θ***_*d*_) (Kino et al., 2009, 2013), which is defined as

$$P={\left(q\left(\theta \right)-q\left(\theta_{d}\right)\right)}^{T}v\left(\theta_{d}\right).$$ (15)

A musculoskeletal system movement converged on a desired joint angle ***θ***_*d*_ when the angle dependent on muscular arrangement, corresponded to a stable equilibrium point in *P* (Kino et al., 2009, 2013). Moreover, the muscular arrangements that were used to attain convergence were identified based on the results of our previous studies (Kino et al., 2009, 2013).

## 4 Joint Stiffness Matrix

The joint stiffness matrix $K_{j}\left(\theta \right)\in R^{2\times 2}$ of the musculoskeletal system (Nakiri and Kino, 2009) is given by

$$K_{j}\left(\theta \right)=-\frac{\partial \tau \left(\theta \right)}{\partial \theta }.$$ (16)

Here, because the joint torque ***τ*** (***θ***) was generated by the MIF controller, the joint stiffness matrix was derived from Eqs 4, 11 as follows:

$$\begin{array}{ccc} \tau \left(\theta \right) & = & W\left(\theta \right)\left(I_{6}-W{\left(\theta_{d}\right)}^{+}W\left(\theta_{d}\right)\right)k_{e}\\ & = & W\left(\theta \right)Y\left(\theta_{d}\right)k_{e}. \end{array}$$ (17)

For convenience, the Jacobi matrix ***W*** (***θ***) from Eq. 3 is rewritten as

$$W\left(\theta \right)=\left[\begin{array}{c} -\frac{\partial q{\left(\theta \right)}^{T}}{\partial \theta_{1}}\\ -\frac{\partial q{\left(\theta \right)}^{T}}{\partial \theta_{2}} \end{array}\right]=\left[\begin{array}{c} w_{a}\left(\theta \right)\\ w_{b}\left(\theta \right) \end{array}\right].$$ (18)

The joint stiffness matrix in Eq. 17 can be re-expressed in terms of its matrix elements as

$$\left[\begin{array}{c} \tau_{1}\left(\theta \right)\\ \tau_{2}\left(\theta \right) \end{array}\right]=\left[\begin{array}{c} w_{a}\left(\theta \right)Y\left(\theta_{d}\right)k_{e}\\ w_{b}\left(\theta \right)Y\left(\theta_{d}\right)k_{e} \end{array}\right].$$ (19)

Equation 19 can then be substituted into Eq. 16 to obtain

$$K_{j}\left(\theta \right)=\left[\begin{array}{cc} -\frac{\partial w_{a}\left(\theta \right)}{\partial \theta_{1}}Y\left(\theta_{d}\right)k_{e} & -\frac{\partial w_{a}\left(\theta \right)}{\partial \theta_{2}}Y\left(\theta_{d}\right)k_{e}\\ -\frac{\partial w_{b}\left(\theta \right)}{\partial \theta_{1}}Y\left(\theta_{d}\right)k_{e} & -\frac{\partial w_{b}\left(\theta \right)}{\partial \theta_{2}}Y\left(\theta_{d}\right)k_{e} \end{array}\right].$$ (20)

Also, from Eq. 18,

$$\frac{\partial w_{a}\left(\theta \right)}{\partial \theta_{2}}=\frac{\partial w_{b}\left(\theta \right)}{\partial \theta_{1}}.$$ (21)

The joint stiffness matrix ***K***_*j*_(***θ***) is a diagonal matrix for which the elements can be obtained using

$$K_{j}\left(\theta \right)=\left[\begin{array}{cc} K_{11}\left(\theta \right) & K_{12}\left(\theta \right)\\ K_{12}\left(\theta \right) & K_{22}\left(\theta \right) \end{array}\right],$$ (22)

from which the following is derived as

$$\left\{\begin{array}{c} K_{11}\left(\theta \right)=-\frac{\partial w_{a}\left(\theta \right)}{\partial \theta_{1}}Y\left(\theta_{d}\right)k_{e}\;\\ K_{12}\left(\theta \right)=-\frac{\partial w_{a}\left(\theta \right)}{\partial \theta_{2}}Y\left(\theta_{d}\right)k_{e}\;\\ K_{22}\left(\theta \right)=-\frac{\partial w_{b}\left(\theta \right)}{\partial \theta_{2}}Y\left(\theta_{d}\right)k_{e}\; \end{array}\right..$$ (23)

In this study, Eq. 23 was used to model two methods of MIF determination. Using the first method, an MIF that eliminates the correlation between the response and stiffness at the desired position was determined. Using the second method, an arbitrary vector describing the stiffness at the initial and desired positions to uniquely determine the balance of MIFs at a given position, was defined.

## 5 Method for Determining Muscular Internal Force Using Joint Stiffness at the Desired Position

In this section, a novel method for determining the MIF, which can eliminate the correlation between the response and stiffness at a desired position, is proposed. Under this method (Method 1), the MIF at the desired position was determined using the joint stiffness.

At a joint angle of ***θ*** = ***θ***_*d*_, the matrix in Eq. 23 becomes

$$K_{r}\left(\theta_{d}\right)=Z\left(\theta_{d}\right)k_{e},$$ (24)

Where

$$\left\{\begin{array}{c} K_{r}\left(\theta_{d}\right)={\left[\begin{array}{ccc} K_{11}\left(\theta_{d}\right), & K_{12}\left(\theta_{d}\right), & K_{22}\left(\theta_{d}\right) \end{array}\right]}^{T},\;\\ Z\left(\theta_{d}\right)=\left[\begin{array}{c} -\frac{\partial w_{a}\left(\theta_{d}\right)}{\partial \theta_{1}}Y\left(\theta_{d}\right)\\ -\frac{\partial w_{a}\left(\theta_{d}\right)}{\partial \theta_{2}}Y\left(\theta_{d}\right)\\ -\frac{\partial w_{b}\left(\theta_{d}\right)}{\partial \theta_{2}}Y\left(\theta_{d}\right) \end{array}\right]\in R^{3\times 6}.\; \end{array}\right.$$ (25)

**FIGURE 2:**
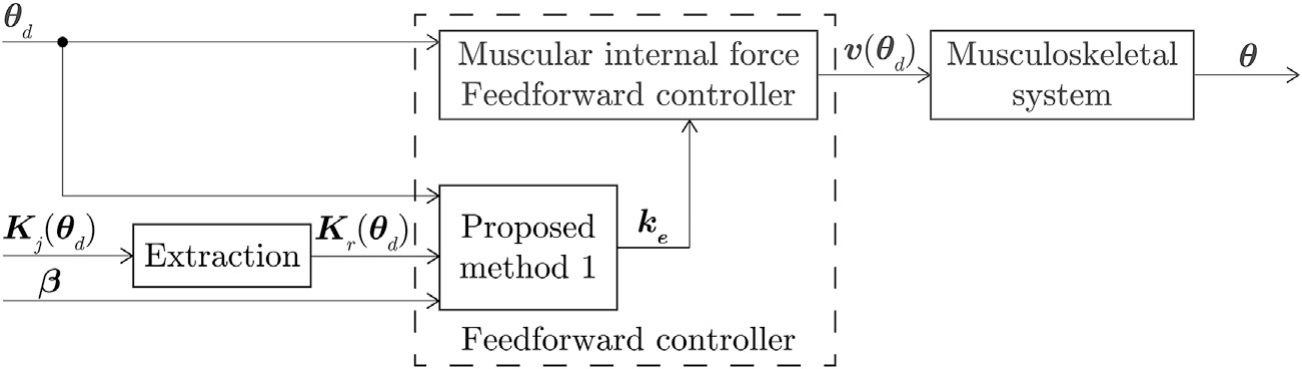
Block diagram of MIF controller using proposed Method 1.

The arbitrary vector ***k***_***e***_ is obtained as the inverse of Eq. 24:

$$k_{e}=Z{\left(\theta_{d}\right)}^{+}K_{r}\left(\theta_{d}\right)+\left(I_{6}-Z{\left(\theta_{d}\right)}^{+}Z\left(\theta_{d}\right)\right)\beta ,$$ (26)

where the matrix $Z{\left(\theta_{d}\right)}^{+}\in R^{6\times 3}$ is a pseudo inverse matrix of the matrix ***Z*** (***θ***_*d*_) defined by

$$Z{\left(\theta \right)}^{+}=Z{\left(\theta \right)}^{T}{\left(Z\left(\theta \right)Z{\left(\theta \right)}^{T}\right)}^{-1},$$ (27)

where $\beta \in R^{6}$ is an arbitrary vector that was set freely within the range satisfying Eq. 12. A block diagram of the application of the MIF controller using Method 1 is shown in Figure 2.

### 5.1 Simulation Results

The simulated positioning control of the musculoskeletal system shown in Figure 1 was conducted to assess the ability of Method 1. This simulation shows that the MIF can eliminate the correlation between the response and stiffness at the desired position using Method 1. In these simulations, an end-point stiffness ellipsoid (Tsuji et al., 1995) was used to obtain a visual representation of the stiffness. Under this approach, the desired stiffness was represented as the end-point stiffness $K_{e}\left(x\left(\theta \right)\right)\in R^{2\times 2}$ , and a joint stiffness ***K***_*j*_(***θ***) corresponding to the end-point stiffness was calculated. The relation between the end-point and joint stiffnesses is given by

$$K_{j}\left(\theta \right)=J{\left(\theta \right)}^{T}K_{e}\left(x\left(\theta \right)\right)J\left(\theta \right).$$ (28)

The MIF can be determined by substituting the elements of the desired joint stiffness, *K*_11_ (***θ***_*d*_), *K*_12_ (***θ***_*d*_), and *K*_22_ (***θ***_*d*_), calculated from the end-point stiffnesses ***K***_*e*_ (***x*** (***θ***_*d*_)), respectively, into Eq. 26.

The results of positioning control using each input determined by three desired end-point stiffness matrixes ***K***_*e*1_ (***x*** (***θ***_*d*_)), ***K***_*e*2_ (***x*** (***θ***_*d*_)), and ***K***_*e*3_ (***x*** (***θ***_*d*_)) were compared in this simulation. The simulation employed a conventional method (CM) (Kino et al., 2013), which minimized the norm of the MIF as a comparison target. The arbitrary vector using CM is given by

$$k_{e}=\gamma {\left[\begin{array}{cccccc} 1.0 & 1.0 & 1.0 & 1.0 & 1.0 & 1.0 \end{array}\right]}^{T}.$$ (29)

The result of the simulation performed by setting *γ* = 70 is expressed as CM1 and that for *γ* = 115 is expressed as CM2. Table 1 lists the physical parameters and muscular arrangements of the musculoskeletal system, respectively, and Table 2 lists the initial and desired parameters. The arbitrary vector ***β*** was set as follows:

$$\beta =150{\left[\begin{array}{cccccc} 1.0 & 1.0 & 1.0 & 1.0 & 1.0 & 1.0 \end{array}\right]}^{T}.$$ (30)

**TABLE 1:** Parameters of musculoskeletal system.

	Parameter	*i* = 1		*i* = 2	
Mass	*m*_*i*_ [kg]	1.678		0.950	
Length	*L*_*i*_ [m]	0.315		0.234	
Moment of inertia	*I*_*i*_ [kgm^2^]	0.011		0.004	
Joint viscosity	*μ*_*i*_ [Ns/rad]	1.0		1.0	

	Parameter	*j* = 1	*j* = 2	*j* = 3	*j* = 4
Muscular arrangement parameter	*a*_*j*_ [mm]	120	120	120	120
	*b*_*j*_ [mm]	20	20	20	20
	*d*_*j*_ [mm]	20	20	20	20
	*h*_*j*_ [mm]	50	50	50	50
	*u*_*j*_ [mm]	50	50	50	50
	*s*_*j*_ [mm]	10	10	10	10

**TABLE 2:** Initial parameters and the results using Method 1.

Parameter	Value
Initial end-point position ***x***_0_	[0.1,0.3]^T^ [m]
Desired end-point position ***x***_*d*_	[−0.2,0.2]^T^ [m]
End-point stiffness matrix ***K***_*e*1_ (***x*** (***θ***_*d*_))	$\left[\begin{array}{cc} 56 & -28\\ -28 & 42 \end{array}\right]$
End-point stiffness matrix ***K***_*e*2_ (***x*** (***θ***_*d*_))	$\left[\begin{array}{cc} 104 & -88\\ -88 & 96 \end{array}\right]$
End-point stiffness matrix ***K***_*e*3_ (***x*** (***θ***_*d*_))	$\left[\begin{array}{cc} 38 & -15\\ -15 & 25 \end{array}\right]$
Joint stiffness vector ***K***_*r*1_ (***x*** (***θ***_*d*_))	${\left[1.680,\;-0.073,\;3.797\right]}^{T}$
Joint stiffness vector ***K***_*r*2_ (***x*** (***θ***_*d*_))	${\left[0.960,\;-0.042,\;9.487\right]}^{T}$
Joint stiffness vector ***K***_*r*3_ (***x*** (***θ***_*d*_))	${\left[1.320,\;-0.121,\;2.244\right]}^{T}$
***k***_***e***_ determined using ***K***_*e*1_ (***x*** (***θ***_*d*_))	${\left[164.9,\;135.2,\;83.0,\;18.4,\;55.6,\;20.9\right]}^{T}$
***k***_***e***_ determined using ***K***_*e*2_ (***x*** (***θ***_*d*_))	${\left[164.9,\;135.2,\;331.7,\;295.8,\;29.4,\;20.9\right]}^{T}$
***k***_***e***_ determined using ***K***_*e*3_ (***x*** (***θ***_*d*_))	${\left[164.9,\;135.2,\;-18.7,\;30.2,\;60.2,\;20.9\right]}^{T}$
Calculation result ***K***_*r*1_ (***x*** (***θ***_*d*_))	${\left[1.680,\;-0.073,\;3.797\right]}^{T}$
Calculation result ***K***_*r*2_ (***x*** (***θ***_*d*_))	${\left[0.960,\;-0.042,\;9.487\right]}^{T}$
Calculation result ***K***_*r*3_ (***x*** (***θ***_*d*_))	${\left[1.320,\;-0.121,\;2.244\right]}^{T}$

Table 2 lists the values of results obtained using Method 1 as determined by Eq. 30.

**FIGURE 3:**
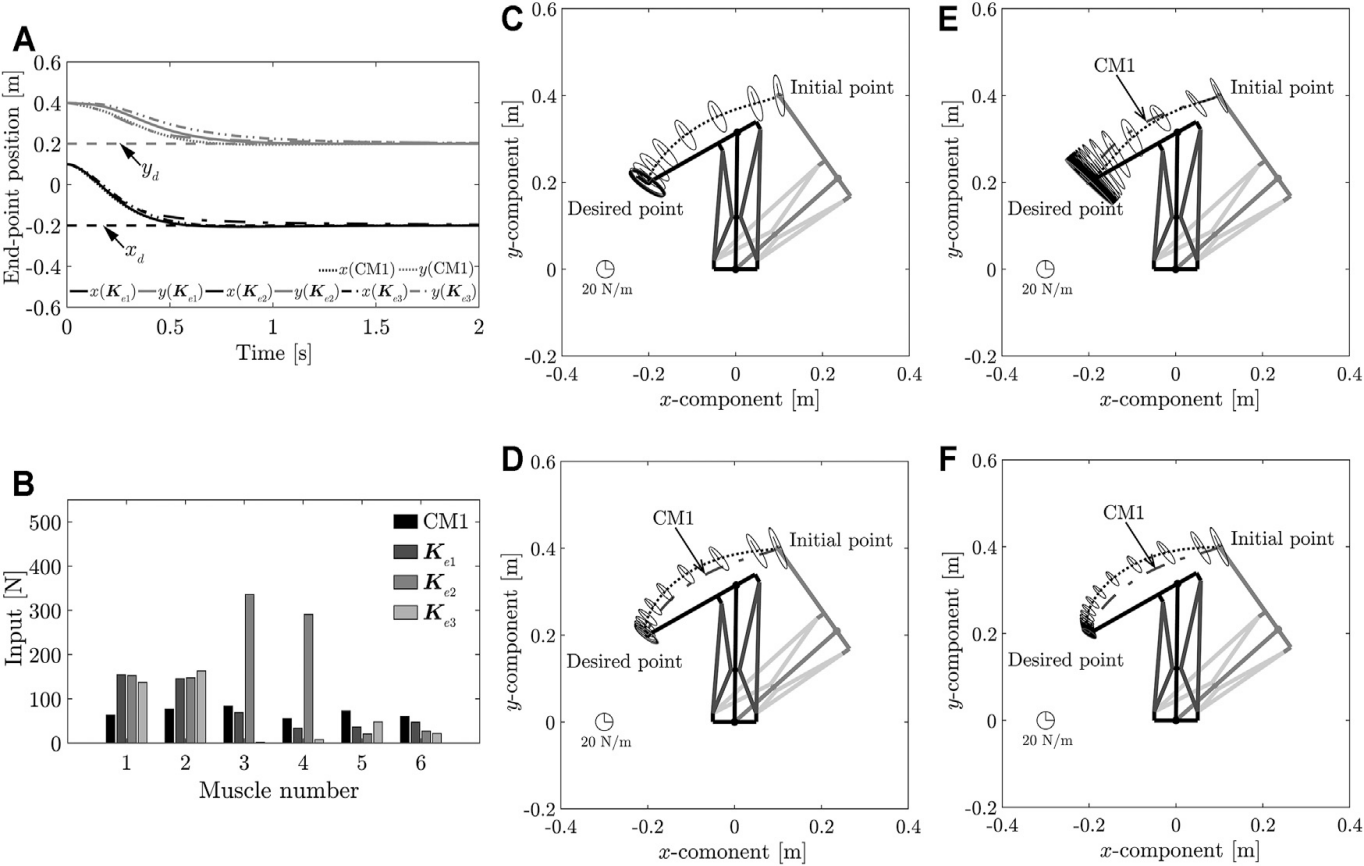
**(A)** Comparison of transient responses of end-point position at different desired end-point stiffnesses. **(B)** Comparison of control inputs. **(C)** Loci of end-point positions and end-point stiffness ellipsoid in the task space produced using CM1. **(D)** Loci of end-point positions and end-point stiffness ellipsoid in the task space produced using the stiffness matrix ***K***_*e*1_. **(E)** Loci of end-point positions and end-point stiffness ellipsoid in task space obtained using the stiffness matrix ***K***_*e*2_. **(F)** Loci of end-point positions and end-point stiffness ellipsoid in task space obtained using the stiffness matrix ***K***_*e*3_.

**FIGURE 4:**
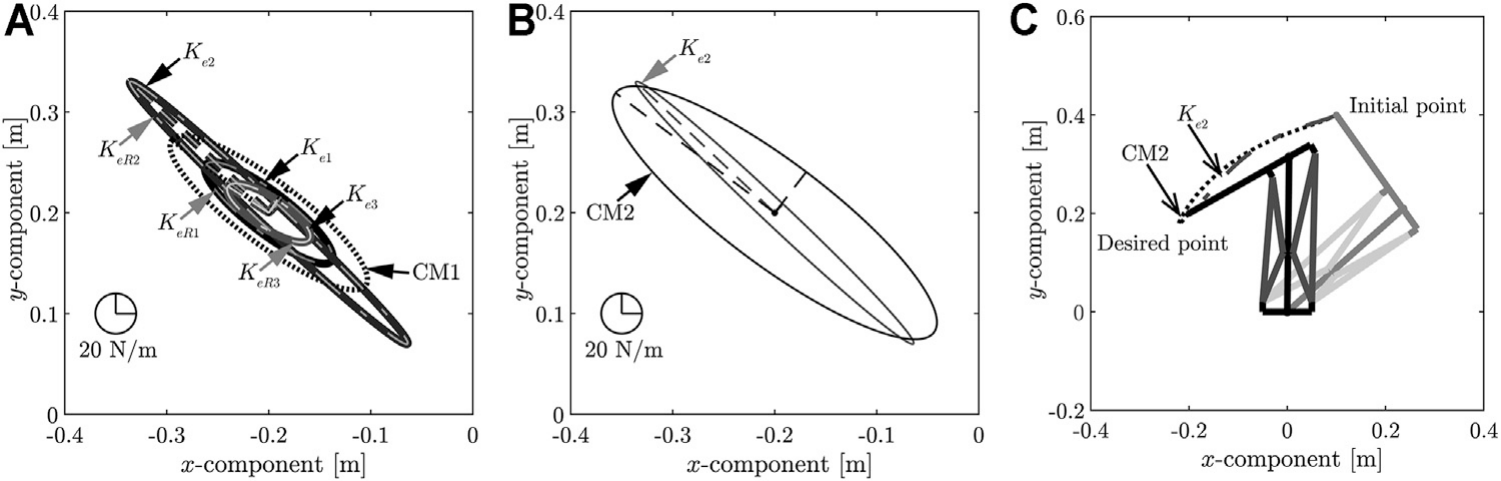
**(A)** Comparison of stiffness ellipsoids obtained using each desired end-point stiffness. **(B)**Comparison of stiffness ellipsoids obtained using the desired end-point stiffness ***K***_*e*2_ and CM2. **(C)** Comparison of loci of end-point positions at the desired end-point stiffnesses ***K***_*e*2_ and CM2.

The results of the simulations are shown in Figure 3 ∼ 4. Figure 3A shows the transient responses of the end-point position; Figure 3B compares the control inputs; Figure 3C ∼ Figure 3F show the end-point stiffness ellipsoids and loci of the end-point positions in the task space, respectively; Figure 4A compares the end-point stiffness ellipsoids; Figure 4B compares the end-point stiffness ellipsoids of CM2 and ***K***_*e*2_ (***x*** (***θ***_*d*_)); Figure 4C compares the loci of the end-point position in the task space of CM2 and ***K***_*e*2_ (***x*** (***θ***_*d*_)). From Figure 3A, Figure 3C ∼ Figure 3F, it is seen that the end-point positions converge at the desired position. The end-point stiffness ellipsoids corresponding to 0.1-s intervals on the end-point trajectories are shown in Figure 3C ∼ Figure 3F. In Figure 3D and Figure 3F, the major axis of the ellipsoid is shortened and the end-point trajectory bulges outward. In Figure 3E, the major axis of the ellipsoid is lengthened and the end-point trajectory suppresses the outward bulge. Although Figure 3F shows an end-point trajectory that is equivalent to the trajectory in Figure 3D, the end-point stiffness ellipsoids at the desired positions differ and the control inputs *v*_*d*3_, *v*_*d*4_ are significantly reduced, as shown in Figure 3B. Those results show that Method 1 can achieve low stiffness without sacrificing the response.

A comparison of the stiffness ellipsoids ***K***_*eR*1_, ***K***_*eR*2_, ***K***_*eR*3_ obtained using the target end-point stiffnesses ***K***_*e*1_, ***K***_*e*2_, ***K***_*e*3_, respectively, is shown in Figure 4A. Figure 4A also shows the stiffness ellipsoids of CM1. In addition, joint stiffness vectors ***K***_*r*_ which were used in determining the MIF, and joint stiffness vectors ***K***_*r*_ which were realized by control, are shown in Table 2. These results depict that the size of the ellipsoid changed, and the desired end-point stiffness used for determining the arbitrary vector ***k***_***e***_ was achieved. However, the ellipsoids showed almost no change in the direction of the major axis. As the direction of the major axis is dependent on the posture of the musculoskeletal system, it is difficult to have a significant impact through the controller input. However, when the arbitrary vector *γ* was set by constructing an end-point stiffness ellipsoid that had the same length as the major axis at the ***K***_*e*2_ ellipsoid, CM2 incurred an overshoot, whereas high stiffness was realized, as shown in Figure 4B and Figure 4C. Method 1 can realize high stiffness without causing an overshoot and can eliminate the correlation between the response and stiffness at the desired position.

### 5.2 Evaluation of End-point Stiffness Ellipsoid

The characteristics of the ellipsoid were evaluated by changing the direction of the major axis using trial and error, to uncover the relation between the end-point stiffness ***K***_*e*_ (***x*** (***θ***_*d*_)) and axis direction, because, as discussed in the preceding section, it is difficult to significantly change the direction of the major axis of the end-point stiffness ellipsoid using control input. The stiffness matrix at the time of inputting the MIF, determined using a conventional method, was used as reference. The elements of the standardized stiffness matrix were changed to rotate the longest axis of the ellipsoid in the clockwise direction. This change was made using trial and error because the relationship between the elements and rotational direction remains unidentified. The MIF calculated based on the stiffness matrix indicates that it satisfied Eq. 12 and was less than 500. If the conditions are satisfied, same elements are changed; otherwise, other elements are modified to rotate the axis in a counter-clockwise direction. Furthermore, the axis length was not evaluated because the lengths of the major and minor axes of the ellipsoid were altered simultaneously while maintaining a constant length ratio. The response of the end-point position was also not considered.

**FIGURE 5:**
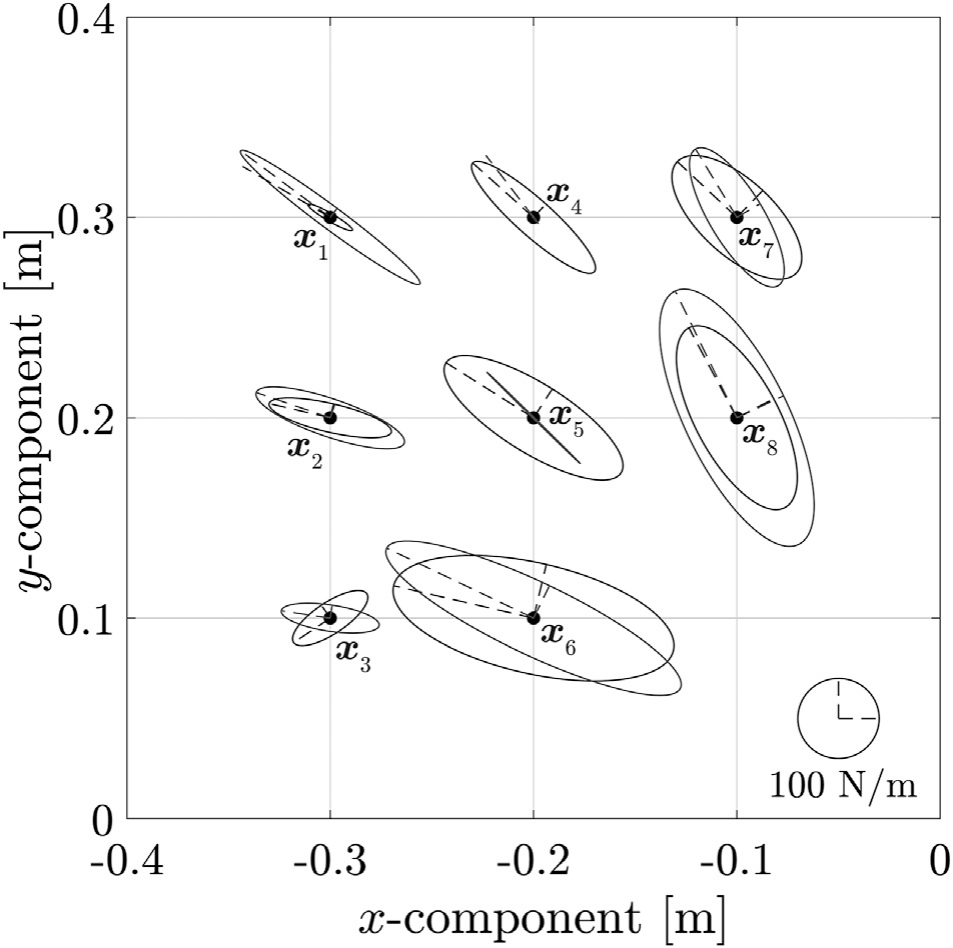
Change in orientation of major axis of ellipsoid with end-point position.

The results of varying the direction of the major axis of the end-point stiffness ellipsoid are shown in Figure 5 and listed in Table 3. It can be observed through the results that the parameters were changed within a range at par with the norm of MIF being less than 500. Figure 5 shows the major axis direction at eight end-point positions. In addition, the end-point position ***x*** = [−0.1,0.1]^T^ was excluded from the evaluation because it was beyond an effective movable range. The minimum change in the angle of *γ* = 2.1° was attained at the position ***x***_8_ = [−0.1,0.2]^T^, while the maximum change in the angle of *γ* = 42.8° occured at ***x***_3_ = [−0.3,0.1]^T^. These results indicate that the direction of the end-point stiffness ellipsoid generated by the application of Method 1 is dependent on the posture of the musculoskeletal system. In this case, the relation between the stiffness matrix parameter ***K***_*e*_ (***x*** (***θ***_*d*_)) and the major axis direction cannot be clarified due to position difference.

**TABLE 3:** Major axis of ellipsoid as a function of end-point position.

End-point position	Rotation angle of Major Axis
***x***_1_ = [−0.3,0.3]^T^	6.5°
***x***_2_ = [−0.3,0.2]^T^	5.8°
***x***_3_ = [−0.3,0.1]^T^	42.8°
***x***_4_ = [−0.2,0.3]^T^	10.6°
***x***_5_ = [−0.2,0.2]^T^	12.2°
***x***_6_ = [−0.2,0.1]^T^	12.6°
***x***_7_ = [−0.1,0.3]^T^	15.7°
***x***_8_ = [−0.1,0.2]^T^	2.1°

### 5.3 Evaluation of Modeling Error

Next, the influence of the musculoskeletal system, including modeling error, was evaluated. The modeling error that occurred during the application of Method 1 to an actual musculoskeletal system could not be ignored. However, the error was justified, as it was difficult to model the actual system accurately. Therefore, the influence of muscular arrangements, including modeling error, was evaluated. In this evaluation, it was assumed that errors in muscular arrangements occurred because of an assembly error in the musculoskeletal system. The proposed method was evaluated as a true value using the parameters listed in Table 1. The evaluation function of the end-point position and joint stiffness vector is defined as

$$\begin{array}{ccc} E_{e} & = & \left\|x_{f}-x_{d}\right\| \end{array}$$ (31)

$$\begin{array}{ccc} E_{k} & = & \|K_{rf}-K_{r}\|, \end{array}$$ (32)

where ***x***_*f*_ is an end-point position of a simulation finish time and ***K***_*rf*_ is the joint stiffness vector at ***x***_*f*_.

One cause for the error in muscular arrangements is the assembly error of the musculoskeletal system. In this case, it was considered that large errors do not occur in the measurement values because muscular arrangements can be easily measured. Therefore, randomly selected values from a range of error rates of 5*%* were used as actual muscular arrangements. The MIF was determined using five muscular arrangements and three stiffness matrices, and the evaluation function was also calculated. Table 4 lists the average values of the five evaluation functions. The evaluation function of the joint stiffness vector was larger than that of the end-point. This is because the joint stiffness vector was calculated using the end-point and muscular arrangement. The evaluation function of the joint stiffness vector became larger because it was difficult to estimate the accurate joint stiffness vector when modeling errors occurred. In particular, the evaluation function of the joint stiffness vector using ***K***_*e*2_ (***x*** (***θ***_*d*_)) was larger than all the other values. This function synergistically became larger because the evaluation function of the end-point was larger than the other values. We propose a robust position control method for the arrangement error at this point (Matsutani et al., 2019a). A decrease in the error of the end-point position as a result of combining the proposed method with the discussed control method is expected. It was assumed that the decrease in the error of the joint stiffness vector was caused by the impact of the end-point position.

**TABLE 4:** Evaluation function of the end-point position and joint stiffness vector.

	*K*_*e*1_ (*x* (*θ*_*d*_))	*K*_*e*2_ (*x* (*θ*_*d*_))	*K*_*e*3_ (*x* (*θ*_*d*_))
Average value of *E*_*e*_	0.047	0.065	0.051
Average value of *E*_*k*_	0.670	2.046	0.654

### 5.4 Evaluation of Arbitrary Vector ***β***

We then investigated the influence of the arbitrary vector ***β*** on the control result. In this evaluation, the MIF was determined using the end-point stiffnesses ***K***_*e*1_ (***x*** (***θ***_*d*_)) listed in Table 2 and the arbitrary vectors ***β*** derived from Eq. 33 ∼ (Eq. 35). The simulation results obtained by applying the resulting MIFs were then compared.

$$\begin{array}{ccc} \beta_{1} & = & 150{\left[\begin{array}{cccccc} 1.0 & 1.0 & 1.0 & 1.0 & 1.0 & 1.0 \end{array}\right]}^{T} \end{array}$$ (33)

$$\begin{array}{ccc} \beta_{2} & = & 5{\left[\begin{array}{cccccc} 1.0 & 1.0 & 1.0 & 1.0 & 1.0 & 1.0 \end{array}\right]}^{T} \end{array}$$ (34)

$$\begin{array}{ccc} \beta_{3} & = & 500{\left[\begin{array}{cccccc} 1.0 & 1.0 & 1.0 & 1.0 & 1.0 & 1.0 \end{array}\right]}^{T}. \end{array}$$ (35)

**FIGURE 6:**
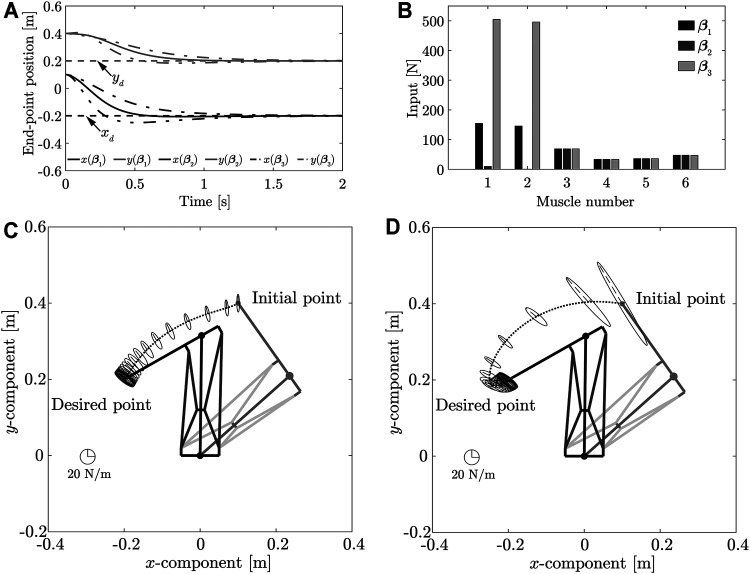
**(A)** Transient response of end-point position as a function of arbitrary vector value. **(B)** Comparison of control inputs obtained using different arbitrary vectors. **(C)** Loci of end-point positions and end-point stiffness ellipsoid in task space obtained using arbitrary vector ***β***_2_. **(D)** Loci of end-point positions and end-point stiffness ellipsoid in task space obtained using arbitrary vector ***β***_3_.

Table 5 lists the arbitrary vectors ***k***_***e***_ derived from application of Method 1, using the outputs of Eq. 33 ∼ (35); the simulation results are shown in Figure 6. Figure 6A shows the transient responses of the end-point position; Figure 6B and Table 5 compare the control inputs; Figure 6C and Figure 6D show the end-point stiffness ellipsoids and loci of end-point positions in the task space, respectively. The results obtained using the arbitrary vector ***β***_1_ were identical to those shown in Figure 3D and were therefore, not repeated. It can be observed from Figure 3D and 6 that, while the stiffness ellipsoids at the desired position were the same, the transient responses of the end-point position differed. While analyzing the changes in end-point stiffness during movement, it was observed that the ellipsoids varied significantly near the initial position, but converged completely near the desired position. This occurs because the arbitrary vector ***β*** affected only the second term of Equation 26, a term that has no effect on the end-point stiffness matrix ***K***_*e*1_ (***x*** (***θ***_*d*_)). This is so, because it becomes a vector that belongs to the null space of the matrix when the end-point position is the desired position. This indicates that Method 1 can be applied to change the transient responses of the end-point position, to achieve the desired end-point stiffness at the same position.

**TABLE 5:** Arbitrary vectors and MIFs determined mathematically using Method 1.

	Arbitrary vector *k*_*e*_
***β***_1_	${\left[164.9,\;135.2,\;83.0,\;18.4,\;55.6,\;20.9\right]}^{T}$
***β***_2_	${\left[5.5,\;4.5,\;110.6,\;-12.0,\;60.9,\;0.7\right]}^{T}$
***β***_3_	${\left[549.8,\;450.6,\;16.3,\;91.5,\;42.8,\;69.5\right]}^{T}$

	MIF ***v*** (***θ***_*d*_)
***β***_1_	${\left[154.6,\;145.5,\;69.0,\;33.6,\;36.1,\;47.3\right]}^{T}$
***β***_2_	${\left[9.5,\;0.5,\;69.0,\;33.5,\;36.1,\;47.3\right]}^{T}$
***β***_3_	${\left[504.6,\;495.7,\;69.2,\;33.9,\;36.0,\;47.1\right]}^{T}$

Changing the arbitrary vector ***β*** from its value in Figure 6B confirms that the arbitrary vector has no effect on the MIFs *v*_*d*3_ ∼ *v*_*d*6_. Similarly, changing the end-point stiffness ***K***_*e*_ (***x*** (***θ***_*d*_)) from its value inFigure 3B confirms that this factor has no significant effect on the MIFs *v*_*d*1_, *v*_*d*2_. These results suggest that muscles 1, 2 contribute to the maintenance of the transient response of the end-point position while muscles 3 ∼ 6 contribute to the maintenance of end-point stiffness. However, determination of the specific roles of the muscles will require further investigation and is left as a subject for future analysis.

## 6 Method of Determining Muscular Internal Force Using Stiffness at the Initial and Desired Positions

In this section, a novel method for uniquely determining the MIF based on the stiffnesses at the initial and desired positions (Method 2) has been introduced. By applying Method 2, the stiffnesses at the respective positions can be controlled while performing repetitive movements. An example of this is the pick-and-place operation, in which the stiffness is set to low when the robot picks up an object, and set to high when the robot places the object.

For a balanced MIF ***v*** (***θ***_0_) input to a muscle at the initial joint angle ***θ***_0_, the joint stiffness ***K***_*j*_ (***θ***_0_) at ***θ***_0_ was derived from Eq. 23 as follows:

$$\left\{\begin{array}{c} K_{11}\left(\theta_{0}\right)=-\frac{\partial w_{a}\left(\theta_{0}\right)}{\partial \theta_{1}}Y\left(\theta_{0}\right)k_{e}\\ K_{12}\left(\theta_{0}\right)=-\frac{\partial w_{a}\left(\theta_{0}\right)}{\partial \theta_{2}}Y\left(\theta_{0}\right)k_{e}\\ K_{22}\left(\theta_{0}\right)=-\frac{\partial w_{b}\left(\theta_{0}\right)}{\partial \theta_{2}}Y\left(\theta_{0}\right)k_{e} \end{array}.\right.$$ (36)

Similarly, for a balance of MIFs ***v*** (***θ***_*d*_) at a desired joint angle ***θ***_*d*_ input to a muscle, the joint stiffness ***K***_*j*_ (***θ***_*d*_) at ***θ***_*d*_ can be derived from Eq. 23 as

$$\left\{\begin{array}{c} K_{11}\left(\theta_{d}\right)=-\frac{\partial w_{a}\left(\theta_{d}\right)}{\partial \theta_{1}}Y\left(\theta_{d}\right)k_{e}\\ K_{12}\left(\theta_{d}\right)=-\frac{\partial w_{a}\left(\theta_{d}\right)}{\partial \theta_{2}}Y\left(\theta_{d}\right)k_{e}\\ K_{22}\left(\theta_{d}\right)=-\frac{\partial w_{b}\left(\theta_{d}\right)}{\partial \theta_{2}}Y\left(\theta_{d}\right)k_{e} \end{array}.\right.$$ (37)

Equations 36 and 37 can be expressed in matrix form as follows:

$$K_{u}=Qk_{e},$$ (38)

where

$$\left\{\begin{array}{c} K_{u}=\left[\begin{array}{c} K_{11}\left(\theta_{0}\right)\\ K_{12}\left(\theta_{0}\right)\\ K_{22}\left(\theta_{0}\right)\\ K_{11}\left(\theta_{d}\right)\\ K_{12}\left(\theta_{d}\right)\\ K_{22}\left(\theta_{d}\right) \end{array}\right]\in R^{6},\\ Q=\left[\begin{array}{c} -\frac{\partial w_{a}\left(\theta_{0}\right)}{\partial \theta_{1}}Y\left(\theta_{0}\right)\\ -\frac{\partial w_{a}\left(\theta_{0}\right)}{\partial \theta_{2}}Y\left(\theta_{0}\right)\\ -\frac{\partial w_{b}\left(\theta_{0}\right)}{\partial \theta_{2}}Y\left(\theta_{0}\right)\\ -\frac{\partial w_{a}\left(\theta_{d}\right)}{\partial \theta_{1}}Y\left(\theta_{d}\right)\\ -\frac{\partial w_{a}\left(\theta_{d}\right)}{\partial \theta_{2}}Y\left(\theta_{d}\right)\\ -\frac{\partial w_{b}\left(\theta_{d}\right)}{\partial \theta_{2}}Y\left(\theta_{d}\right) \end{array}\right]\in R^{6\times 6}. \end{array}\right.$$ (39)

The arbitrary vector ***k***_***e***_ can be obtained as the inverse of Eq. 38 as follows:

$$k_{e}=Q^{-1}K_{u}.$$ (40)

**FIGURE 7:**
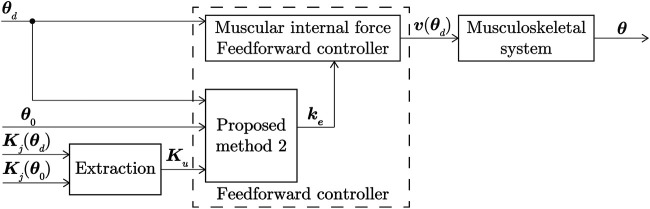
Block diagram of application of Method 2 using MIF controller.

The MIFs ***v*** (***θ***_0_) and ***v*** (***θ***_*d*_) calculated using this arbitrary vector correspond to the musculoskeletal system stiffening to values of ***K***_*j*_ (***θ***_0_) and ***K***_*j*_ (***θ***_*d*_) at positions ***θ***_0_ and ***θ***_*d*_, respectively. The application of Method 2 using the MIF controller is shown as a block diagram in Figure 7.

### 6.1 Simulation Results

Method 2 was used to apply individual control inputs to the musculoskeletal system as it carried out repetitive movements. By changing the values of the arbitrary vector ***k***_***e***_, different target end-point stiffnesses were applied at various initial positions *A*, to serve as control inputs to the muscle for moving the system from *A* to a desired position *B*. Arbitrary values of ***k***_***e***_ were also applied as desired end-point stiffnesses at *B*, using the method. The simulated positioning control of the musculoskeletal system shown in Figure 1 was conducted to evaluate the abilities of Method 2. A simulation was performed to infer that the MIF determined using Method 2, can realize the stiffness at two positions. Table 6 lists the arbitrary vector determined using Method 2 and the set of initial and desired positions, *A* and *B*, used in the simulations, respectively.

**TABLE 6:** Position *A* and *B* parameters and the arbitrary vector determined using Method 2.

End-point position *A*	*x*_*A*_ = [0.0,0.4]^T^ [m]
End-point position *B*	***x***_*B*_ = [−0.2,0.2]^T^ [m]
	$K_{eA}=[\begin{array}{cc} 25 & -22\\ -22 & 61 \end{array}]$
End-point stiffness matrix	$K_{eB}=[\begin{array}{cc} 142 & -61\\ -61 & 102 \end{array}]$
Calculation result	$k_{e}={\left[1068.0,\;-845.4,\;-669.7,\;884.4,\;447.0,\;241.0\right]}^{T}$

In each simulation, the musculoskeletal system underwent controlled movement from the initial position *A* to the desired position *B* and then back from *B* to *A*. The results validated the effectiveness of Method 2 in terms of controlling the musculoskeletal system based on control of stiffness at the end-point and other positions. The physical and muscular parameters of the musculoskeletal system used in this simulation are listed in Table 1.

#### 6.1.1 Position Control for Moving From Position *A* to *B*

**FIGURE 8:**
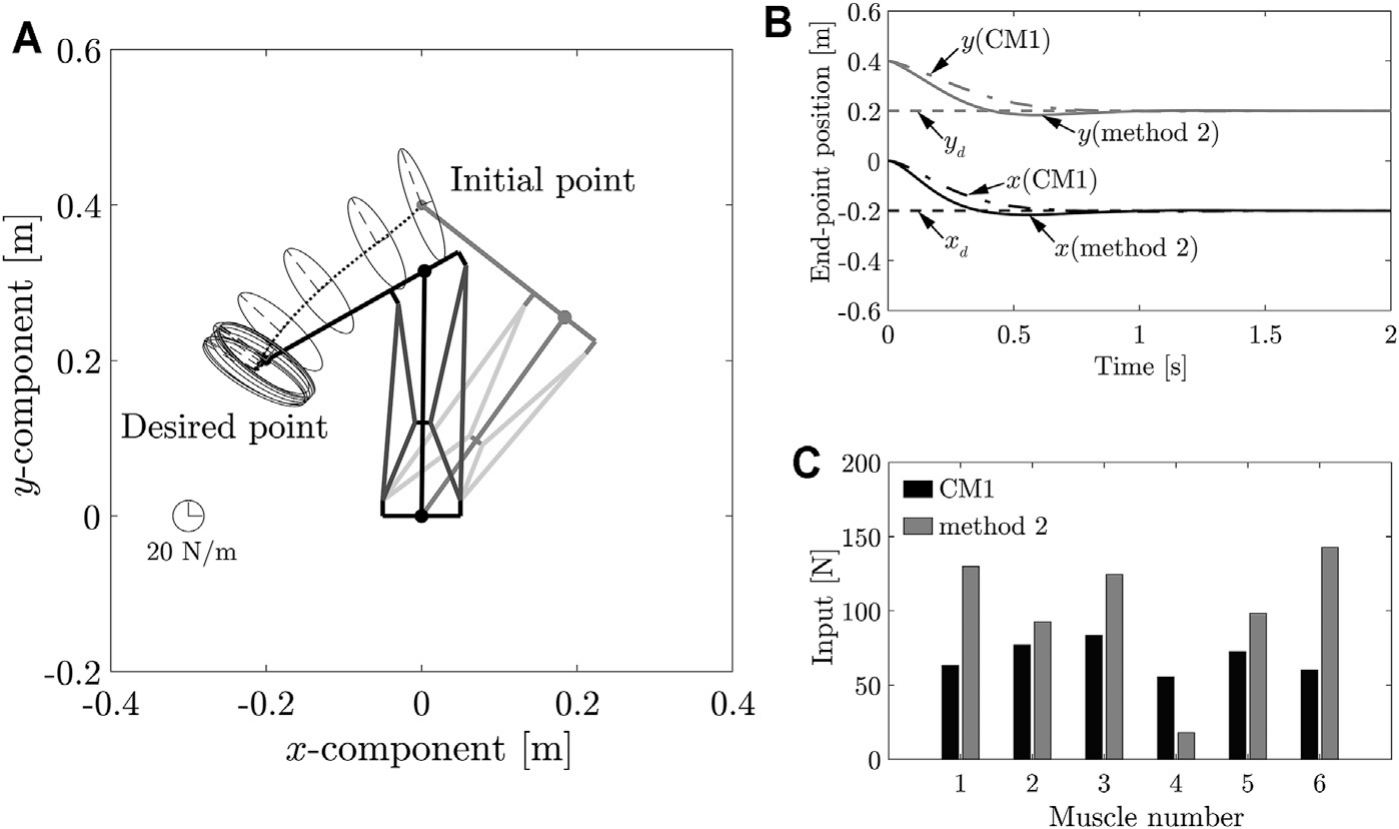
**(A)** Loci of end-point positions during movement from positions *A* to *B*, and end-point stiffness ellipsoids in task space. **(B)** Transient response of end-point position during movement from position *A* to position *B*. **(C)** Control input using MIF balancing at position *A*.

Further, the position control process for motion between positions *A* and *B* was then simulated. The results of the simulation are shown in Figure 8. Figure 8A shows the end-point stiffness ellipsoids and loci of the end-point position in the task space; Figure 8B shows the transient responses of the end-point position; Figure 8C compares the respective control inputs. It can be observed from Figure 8 that the end-point position converged at the desired position ***x***_*B*_, and that end-point stiffness ***K***_*eB*_ was achieved at the desired position ***x***_*B*_. As shown in Equation 36, a balanced MIF of ***v*** (***θ***_*A*_) at the initial position was required to achieve an end-point stiffness of ***K***_*eA*_ at this position. The end-point stiffness at the initial position ***x***_*A*_ in Figure 8A differed from the end-point stiffness ***K***_*eA*_ because the position control applied a balanced MIF ***v*** (***θ***_*B*_) at the desired position ***x***_*B*_. The control input *v*_*dl*_ determined by Method 2 (Figure 8C) satisfied Eq. 12.

#### 6.1.2 Position Control for Moving From Position *B* to *A*

**FIGURE 9:**
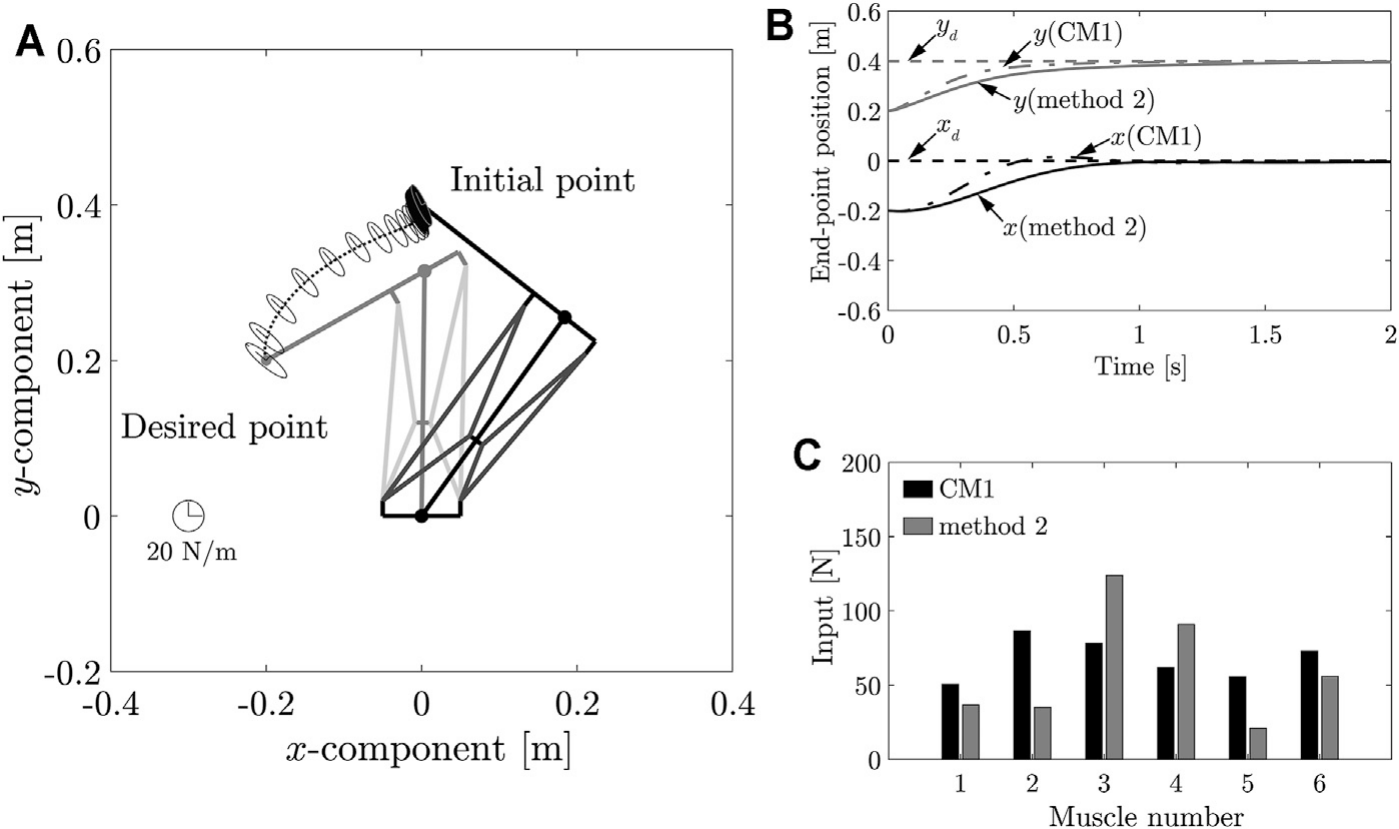
**(A)** Loci of end-point positions during movement from positions *B* to *A* and end-point stiffness ellipsoids in task space. **(B)** Transient response of end-point position during movement from position *B* to position *A*. **(C)** Control input using MIF balancing at position *A*.

We then simulated position control for the movement from position *B* to *A*. The results of this simulation are shown in Figure 9. Figure 9A shows the end-point stiffness ellipsoids and loci of the end-point positions in the task space. Figure 9B shows the transient responses of the end-point position. While, Figure 9C compares the control inputs. It can be observed from Figure 9 that the end-point position converged at the desired position ***x***_*A*_ and that an end-point stiffness ***K***_*eA*_ was achieved at the desired position. The control input determined by applying Method 2 (Figure 9C) satisfied Eq. 12. The results in this section and the preceding one demonstrate that Method 2 can be applied to uniquely determine the MIF. It is difficult to significantly alter the direction of the major axis of the end-point stiffness ellipsoid using the control input because the end-point stiffness depends on the posture of the musculoskeletal system. However, this method can still be used to control the end-point stiffnesses at the two positions effectively. In this study, the control input can be uniquely determined using Method 2 because the control target is a two-link system with six muscles. If Method 2 were used in a system with a greater number of redundancies, it would not be possible to determine the control input uniquely. Meanwhile, it was considered to increase the selectable range of the direction of the end-point stiffness ellipsoid. In addition, the control input can be uniquely determined by setting subtasks according to the redundancies in this case.

## 7 Conclusion

In this study, two new methods for determining MIF using joint stiffness were developed, to solve the issues in a previously developed musculoskeletal system MIF feedforward controller. Using the first method (Method 1), a MIF that eliminates the correlation between the response and stiffness at a desired position was determined. Using the second method (Method 2), an arbitrary vector that reflects the stiffnesses at the initial and desired positions was determined, and applied to uniquely determine the balance of MIFs at each position. Although it was found to be difficult to significantly alter the stiffness using the control input, as the end-point stiffness depends on the posture of the musculoskeletal system, we believe that the proposed system can be adapted to expand the region of feasible stiffness control (Matsutani et al., 2017); and this will be a subject of our future analysis. The proposed methods can realize sensorless position and stiffness control. In addition, by combining the existing control method with the proposed method, runaway of movement at sensor crashes can be prevented. Further, we plan to experimentally verify this in a future study.

Furthermore, the MIF is expected to be combined with feedback control in a future work. This paper describes the evaluation of the musculoskeletal system, including the modeling error. Feedforward control is incapable of handling unanticipated contact. A combination of the MIF and feedback control complement each other to improve accuracy. For example, a stable region of feedback control that tends to become unstable can be assured by combining feedforward control based on the potential no needing an inverse dynamics model (Matsutani et al., 2018). In future works, this combination needs to be discussed in detail.

## Data Availability

The original contributions presented in the study are included in the article/Supplementary Material, further inquiries can be directed to the corresponding author.
